# Clinical utility of MRI in the decision-making process before radical prostatectomy: Systematic review and meta-analysis

**DOI:** 10.1371/journal.pone.0210194

**Published:** 2019-01-07

**Authors:** Mieszko Kozikowski, Wojciech Malewski, Wojciech Michalak, Jakub Dobruch

**Affiliations:** Department of Urology, Centre of Postgraduate Medical Education, Warsaw, Poland; Imperial College London, UNITED KINGDOM

## Abstract

**Context:**

Magnetic resonance imaging (MRI) is currently the most accurate imaging modality to assess local prostate cancer stage. Despite a growing body of evidence, incorporation of MRI images into decision-making process concerning surgical template of radical prostatectomy, is complex and still poorly understood.

**Objective:**

We sought to determine the value of MRI in preoperative planning before radical prostatectomy.

**Materials and methods:**

Systematic search through electronic PubMed, EMBASE, and Cochrane databases from 2000 up to April 2018 was performed. Only studies that used preoperative MRI in decision-making process regarding extension of resection in patients with prostate cancer, in whom radical prostatectomy was an initial form of treatment were included into analysis. Their quality was scored by Risk Of Bias In Non-Randomized Studies of Interventions system. Meta-analysis was performed to calculate the weighted summary proportion under the fixed or random effects model as appropriate and pooled effects were depicted on forest plots.

**Results:**

The results showed that the preoperative MRI led to the modification of initial surgical template in one third of cases (35%). This occurred increasingly with the rising prostate cancer-risk category: 28%, 33%, 52% in low-, intermediate- and high-risk group, respectively. Modification of neurovascular bundle-sparing surgery based on MRI appeared to have no impact on the positive surgical margin rate. The decision based on MRI was correct on average in 77% of cases and differed across prostate cancer-risk categories: 63%, 75% and 91% in low-, intermediate- and high-risk group, accordingly.

**Conclusions:**

In summary, MRI has a considerable impact on the decision-making process regarding the extent of resection during radical prostatectomy. Adaptation of MRI images by operating surgeons has at worst no significant impact on surgical margin status, however its ability to decrease the positive surgical margin rates remains unconfirmed.

## Introduction

Radical prostatectomy (RP) remains the mainstay therapy of organ confined prostate cancer (PCa) [1]. Its oncological efficacy has been well established throughout the recent years [2,3]. However, morbidity associated with RP including incontinence and erectile dysfunction is very common and should be a matter of preoperative counselling. To avoid unfavorable consequences, urologists aim to restrict their surgical templates and spare neurovascular bundles (NVBs) and improve apical dissection, and yet provide negative surgical margins. The latter, together with undetectable postoperative prostate-specific antigen (PSA) at 3 months after surgery are recognized surrogate of oncological outcome [4].

Preservation of NVBs has been shown to foster erectile function recovery [5]. Moreover, a correlation between the extent of NVBs resection and postoperative urinary continence has been acknowledged [6], therefore NVB-sparing should be attempted whenever possible to maintain quality of life. Currently, EAU-ESTRO-ESUR-SIOG recommendations encourage to perform nerve-sparing surgery in patients with a low risk of extracapsular disease and conversely, establish clear contraindications in case of a high risk of extracapsular disease, such as any cT2c or cT3 PCa, and any Gleason score (GS) > 7 on biopsy [1].

Magnetic resonance imaging (MRI) is currently the most accurate imaging modality that provides relevant information on PCa localization and stage [7]. However, in spite of a growing body of evidence, influence of MRI on decision-making process, with adjustment of individual template of dissection during subsequent RP is complex and still poorly understood. EAU-ESTRO-ESUR-SIOG guidelines suggest using prostate MRI for local staging in high risk group and intermediate risk group with predominant Gleason pattern 4 [1], that is in those, in whom NVB sparing approach should be avoided. Conversely, in randomized trial preoperative MRI has been associated with reduction of positive surgical margins in low risk PCa only [8]. Understandably, the different prevalence of EPE in risk stratified cohorts highly influence the diagnostic performance of MRI [9]. For example, the low risk patients would benefit the most from the ability of MRI to exclude EPE by selecting right candidates for NVB-sparing surgery. Conversely, in high-risk patients the role of MRI is to detect tumor infiltration beyond the capsule as knowledge of the site of EPE might help in reducing the substantial risk of PSM. Therefore, we sought to determine the value of MRI in preoperative planning prior to RP with specific focus on the attitude towards extent of neurovascular bundles removal.

## Evidence acquisition

### Protocol registration

The protocol of this review was registered in the Prospective Register of Systematic Review (PROSPERO number: CRD42017060064) under the working title: "Influence of preoperative MRI on a decision-making process prior radical prostatectomy” (http://www.crd.york.ac.uk/PROSPERO/display_record.php?ID=CRD42017060064). The study was carried out according to the Preferred Reported Items for Systematic Reviews and Meta-analysis guidelines (PRISMA, http://www.prisma-statement.org).

### Data search

A systematic literature search was performed in following databases: MEDLINE via PubMed, Embase via Ovid and the Cochrane Database of Systematic Reviews. The search was restricted to publications in English, dating from January 2000 to April 2018. Over the past two decades there has been a huge improvement in prostate MRI as a result of a combination of high magnetic field strength and multiparametric imaging technique as well as advancements in the standardized interpretation of images. For these reasons we restricted our search form 2000 onwards [7]. Following search terms and their abbreviations were used in all databases: ("prostatic neoplasms” OR "prostate cancer” OR "prostate” OR „prostatic") AND ("magnetic resonance imaging" OR "multiparametric magnetic resonance imaging") AND ("prostatectomy" OR "radical prostatectomy" OR "laparoscopic radical prostatectomy" OR "endoscopic radical prostatectomy" OR "open radical prostatectomy" OR "robot-assissted laparoscopic prostatectomy”). The Mendeley Desktop version 1.17.9 (2008–2016 Mendeley Ltd.) citation manager was used to store records and remove duplicates. Reference lists of included papers and latest review articles were also hand-searched. To provide a completeness of this review, additional papers covering the subject and published during the current year were also searched.

The primary research question for this systematic review is: how does the preoperative MRI influence a decision-making process before RP? Eligible studies were those with study cohort of men diagnosed with PCa confirmed on biopsy, evaluated preoperatively with MRI in whom RP was an initial form of treatment. Pathological examination of prostatectomy specimen with respect to PCa stage and postoperative margin status was the reference standard. Special attention was devoted to publications where the decision-making process regarding extension of resection based on MRI was explicitly described.

**Fig 1:**
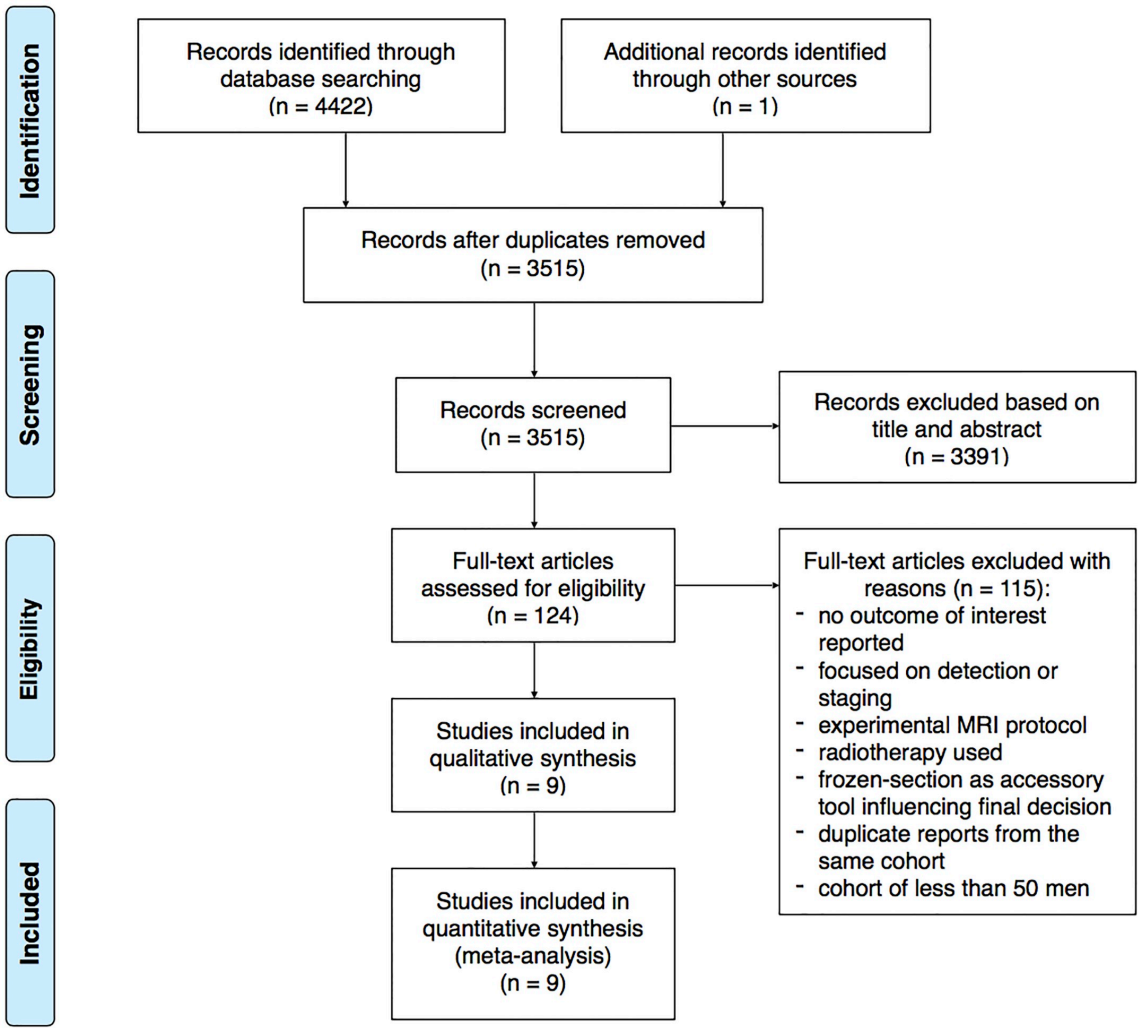
Flow chart of the study inclusion process according to PRISMA.

Three investigators (M.K. W.M. and W.M.) independently screened and assessed the eligibility of the articles based on their title and abstract. Any disagreements were resolved by unanimous decision and accepted by the senior author (J.D.). Subsequently full text articles were reviewed and included in the systematic review or excluded with certain reasons. The data extraction form was designed according to PRISMA (Fig 1). Due to heterogenous study designs and variety of assessed outcomes, extracted data appeared to be suitable for meta-analysis of 9 studies (Tables 1 and 2) [8,10,11,12,13,14,15,16,17]. The 115 studies not fulfilling the inclusion criteria were excluded for following reasons: not reporting the outcomes of interest; being focused only on PCa detection or staging; including experimental MRI protocol; using radiotherapy; using frozen-section as accessory tool influencing final decision; being duplicate reports from the same cohort; comprising cohort of less than 50 men. A total of 1552 men were included with a cohort size ranging from 75 to 353.

**Table 1:** Basic characteristics of studies included into systematic review and meta-analysis.

Study			Study characteristics			Patient characteristics		
First author	Year	Type	Study size	Study design	Subgroup analysis	Mean age [years]	Mean preoperative PSA [ng/ml]	Mean biopsy Gleason score
H. Hricak [10]	2004	full article	135	prospective cohort	Y	58	5.8	6
A.P. Labanaris [11]	2009	full article	75	retrospective cohort	N	58	9.9	6
T.D. McClure [12]	2012	full article	104	prospective cohort	N	60	6.5	6
V. Panebianco [13]	2012	full article and conference abstract	125	prospective cohort	N	57	5.7	6
B.H. Park [14]	2014	full article and conference abstract	353	retrospective cohort	Y	64	5.3	6
E. Rud [8]	2015	full article and conference abstract	222 MRI, 216 non-MRI	randomized controlled trial	Y	62	7.8	7
J.P. Radtke [15]	2015	full article	132	retrospective cohort	Y	66	8.2	-
R. Schiavina [16]	2017	full article	137 MRI, 166 non-MRI	prospective cohort	N	64	9.7	7
M. Kozikowski [17]	2018	full article	154	retrospective cohort	Y	63	10.6	7

MRI magnetic resonance imaging.

**Table 2:** Imaging and surgical characteristics of studies included into systematic review and meta-analysis.

Study	Imaging characteristics				Surgery specification	NVB-sparing surgery	Staging results	
First author	Field strength [Tesla]	(mp)MRI: sequences	Endorectal coil	Time of MRI			Sensitivity	Specificity
H. Hricak [10]	1.5	MRI: T2WI	Y	preoperative	RRP	83% NVB*	-	-
A.P. Labanaris [11]	1.0	mpMRI: T2WI, DWI, DCE	N	preoperative	RRP	79%	92%	100%
T.D. McClure [12]	1.5	mpMRI: T2WI, DWI, DCE, MRSI	Y	preoperative	RALP	85% NVB*	50%	98%
V. Panebianco [13]	3.0	mpMRI: T2WI, DWI, DCE, MRSI, DTI	Y	preoperative	RRP	91%	-	-
B.H. Park [14]	3.0	mpMRI: T2WI, DWI, DCE	N	preoperative	RALP	79%	56%	82%
E. Rud [8]	1.5	mpMRI: T2WI, DWI	N	preoperative	RALP	31% NVB*	73%	65%
J.P. Radtke [15]	3.0	mpMRI: T2WI, DWI, DCE	N	pre-biopsy	RRP, RALP	75%	-	-
R. Schiavina [16]	1.5 or 3.0	mpMRI	Y/N	preoperative	RALP	81%	-	-
M. Kozikowski [17]	3.0	mpMRI: T2WI, DWI, DCE	N	preoperative	LRP	71%	41%	93%

(mp)MRI (multiparametric) magnetic resonance imaging; T2WI T2-weighted imaging; DWI diffusion-weighted imaging; DCE dynamic contrast-enhanced imaging; MRSI magnetic resonance spectroscopic imaging; DTI diffusion tensor imaging; RRP retropubic radical prostatectomy; RALP robot-assisted laparoscopic prostatectomy; LRP laparoscopic radical prostatectomy; NVB neurovascular bundle.

* Analysis performed on side, not on patient basis.

R program (version 3.2.3, the R foundation for Statistical Computing, http://www.r-project.org) with meta and metafor packages was used to perform statistical analysis. Heterogeneity of the studies was assessed using *I*^*2*^ statistics and in case of significant heterogeneity (*I*^*2*^ > 50%), random effects model was favored over fixed effect model. Weighted summary proportions were calculated pooled effects were depicted on forest plots.

### Risk of bias assessment

Nine studies were included in the final analysis. Methodologic quality was assessed using "Risk Of Bias In Non-randomized Studies of Interventions" (ROBINS-I) scoring system, which is a new tool for evaluating risk of bias [18]. ROBINS-I views each study as an attempt to simulate an ideal randomized trial, that is expected to answer a particular clinical problem. Seven domains are investigated for potential risk of introducing bias, that are judged with use of signaling questions.

**Fig 2:**
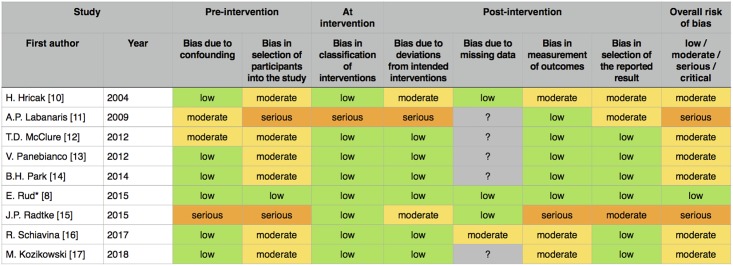
The risk of bias assessment of included papers using ROBINS-I tool for non-randomized studies. *randomized clinical trial.

Overall, the risk of bias was moderate in most papers, which is understandable as most studies were non-randomized and had the retrospective design, and as such are subject to confounding and a range of other biases (Fig 2). At the pre-intervention stage, bias due to confounding variables was mainly low, except of three cases, of which in one study serious bias was found because surgeons were not aware of MRI results. Moderate selection bias found in most of the included studies reflects the lack of randomization and control groups. Moreover, highly selective inclusion criteria were spotted in two papers where patients with evidence of EPE or very-high risk PCa were excluded from analysis. Only one study was found to have a serious risk of bias at intervention, because it investigated a presumptive influence of MRI, if it had been implemented. For the same reason this paper was assessed to be seriously biased due to non-adherence to intended intervention. Bias due to missing information about postoperative status of patients was not clearly stated in five studies. Outcomes of interest (NVB-sparing, the PSM rate, the appropriateness of decision) were reported incompletely in four papers, of which one had several of these shortcomings. Biased selection of reported results concerned three papers, in which important outcomes were reported incompletely, only for the part of cohort, which precluded the estimates from being used in our meta-analysis. To date, only one randomized clinical trial concerning the subject of this review was published, however it was assessed with the same tool to maintain coherence. The quality assessment was performed by two independent reviewers.

## Evidence synthesis

Main characteristics of studies included into systematic review and meta-analysis are presented in Tables 1 and 2. Outcomes of interest extracted from eligible studies are listed in Table 3.

**Table 3:** Data extracted from eligible studies for meta-analysis.

Study	Decision on NVB-sparing				NVB-sparing technique					PSM
First author	Decision unchanged	Decision changed	More aggressive NVB resection	More preserving NVB-sparing	Any NVB-sparing	Bilateral NVB-sparing	Unilateral NVB-sparing	Partial NVB-sparing	Non-sparing	
H. Hricak [10]	61% NVB*	39% NVB*	25% NVB*	14% NVB*	83% NVB*	-	-	19% NVB	17% NVB	-
A.P. Labanaris [11]	66%	44%	15%	29%	79%	77%	1%	-	16%	-
T.D. McClure [12]	73%	27%	11%	17%	85% NVB*	-	-	-	15% NVB*	7%
V. Panebianco [13]	70%	30%	30%	0%	91%	74%	17%	-	9%	8%
B.H. Park [14]	74%	26%	11%	15%	78%	57%	21%	-	22%	13%
E. Rud [8]	63%	27%	27%	0%	31% NVB	9%	11%	11%	69% NVB*	19%
J.P. Radtke** [15]	69%	31%	18%	13%	75%	-	-	-	25%	27%
R. Schiavina [16]	53%	47%	55%	45%	81%	56%	25%	-	19%	12%
M. Kozikowski [17]	55%	45%	34%	11%	71%	21%	50%	-	29%	15%

NVB neurovascular bundle; PSM positive surgical margin.

* Analysis performed on side, not on patient basis.

** Hypothetical data on NVB-sparing and PSM.

### Impact of MRI on NVB-sparing surgery

**Fig 3:**
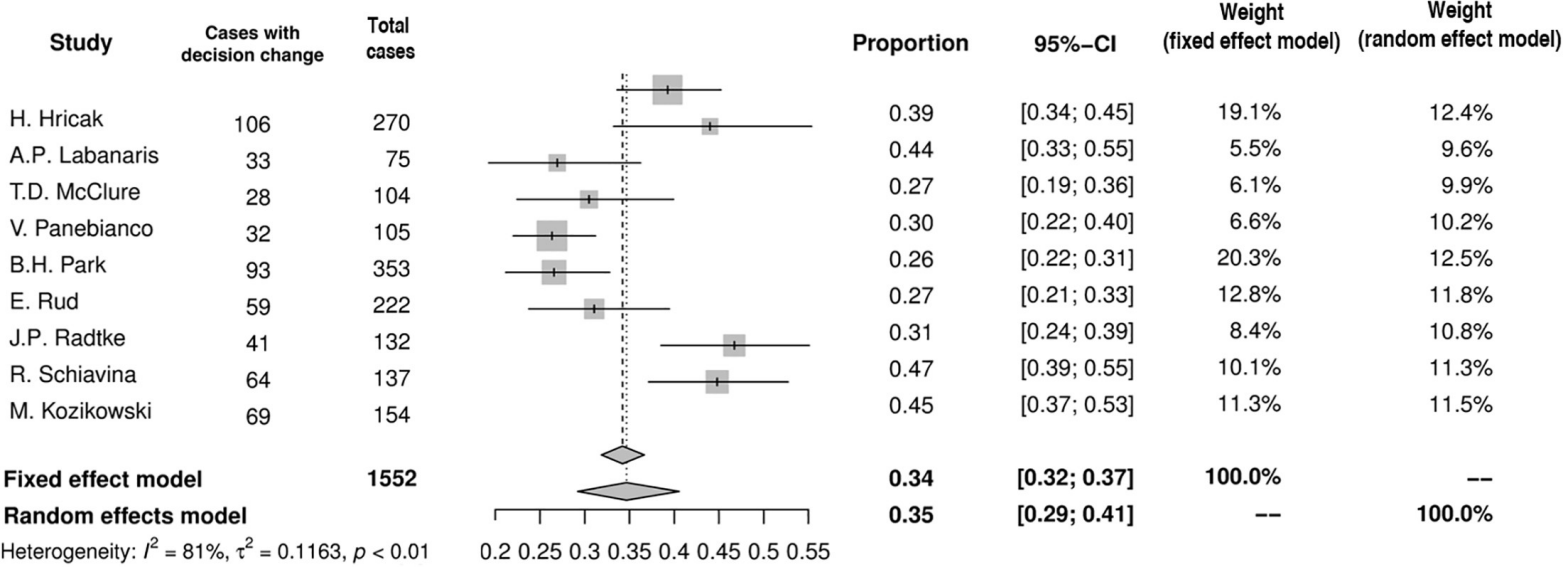
Forest plot of proportions showing decision change in overall.

**Fig 4:**
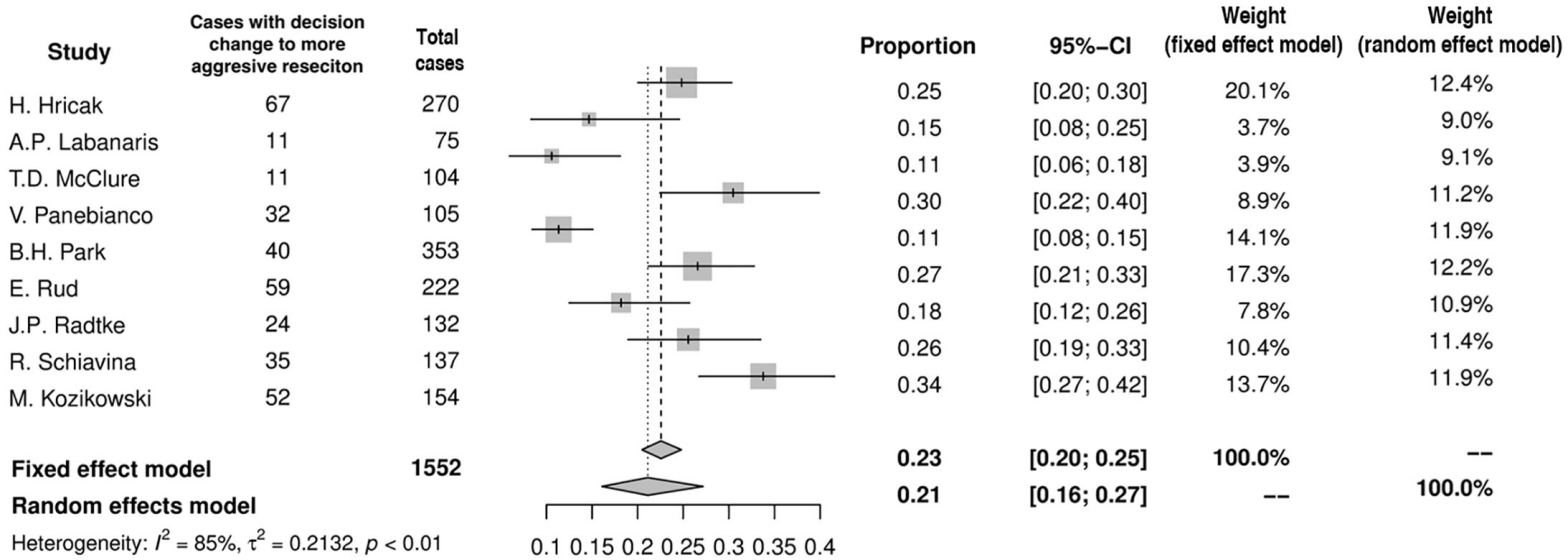
Forest plot of proportions showing decision change to more aggressive resection.

**Fig 5:**
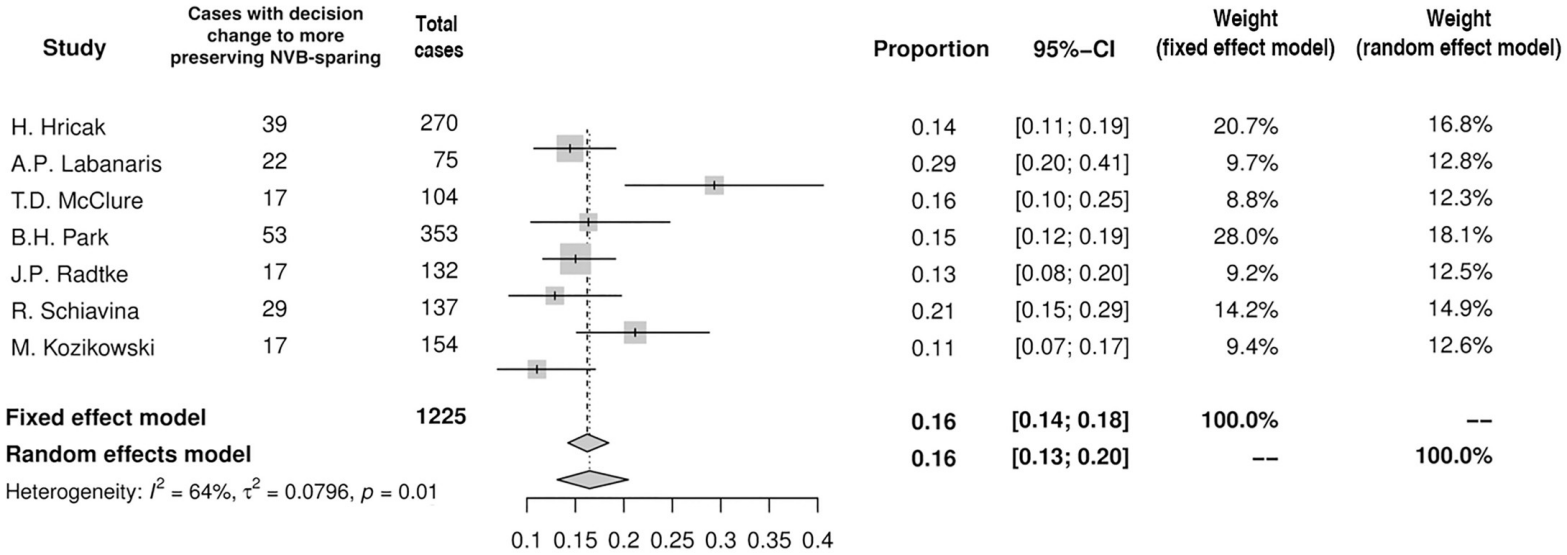
Forest plot of proportions showing decision change to more preserving NVB-sparing.

Based on preoperative MRI the decision regarding NVB-sparing was changed in 35% (95%CI: 29–41%) of men (n = 525) in the summary of proportions in ten studies included into analysis (Fig 3). In most studies the decision tended to be modified in either of two ways: more aggressive resection (n = 331) or more preserving NVB-sparing (n = 194). In pooled analysis more aggressive resection was chosen in 21% (95%CI: 16–27%) of men (Fig 4), whereas more preserving NVB-sparing was preferred in 16% (95%CI: 13–20%) of patients (Fig 5). Moreover, in two studies MRI altered the surgical plan exclusively in the direction of more radical excision [8,13].

### Differential influence of MRI imaging across PCa risk groups

**Fig 6:**
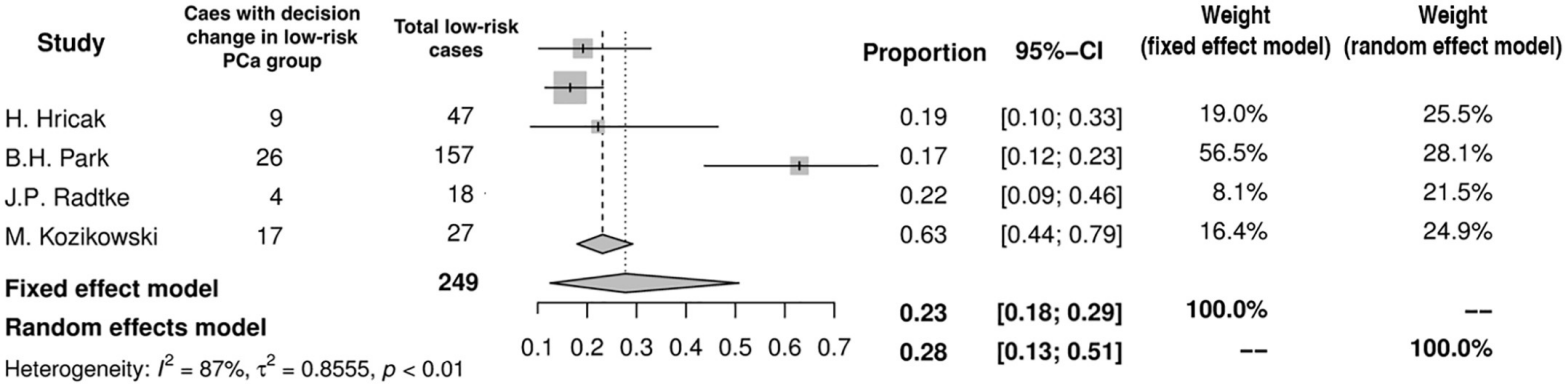
Forest plot of proportions showing decision change in low-risk PCa group.

**Fig 7:**
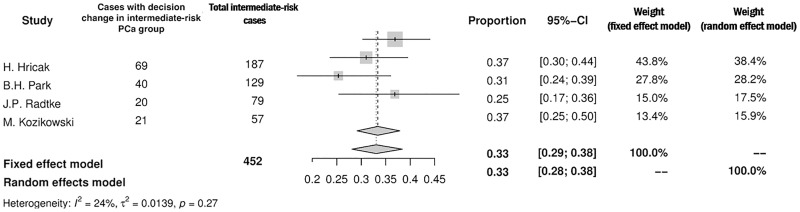
Forest plot of proportions showing decision change in intermediate-risk PCa group.

**Fig 8:**
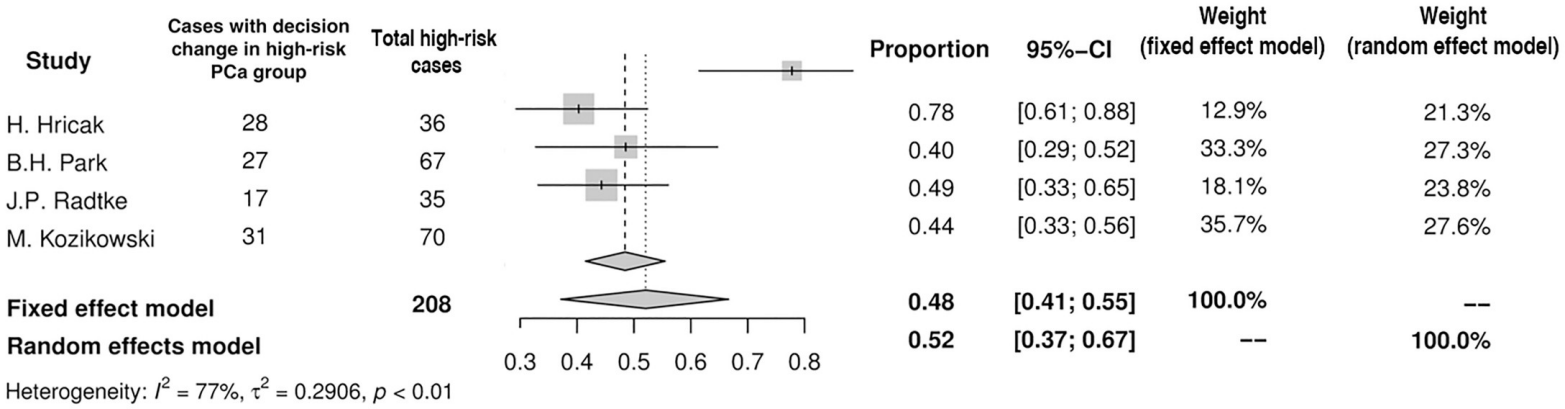
Forest plot of proportions showing decision change in high-risk PCa group.

The rates of patients in whom the surgical plan regarding NVB was modified after MRI varied significantly among PCa-risk groups. Only 4 studies provided sufficient data with this respect. In the low-risk PCa group (n = 249) the decision was changed in 28% (95%CI: 13–51%) exclusively toward more aggressive resection (Fig 6). In the intermediate- and high-risk PCa group the corresponding value was 33% (95%CI: 29–38%) and 52% (95%CI: 37–67%), respectively (Figs 7 and 8). However, in the intermediate-risk PCa group the decision was modified in 19% (95%CI: 10–32%) of men to more aggressive resection and in 14% (95%CI: 7–29%) of men to more preserving surgery (results not displayed on a forest plot). In the high risk PCa group MRI resulted in more preserving template in 31% (95%CI: 21–43%) and with more aggressive one in 25% (95%CI: 12–45%).

## PSM rate and decision-making process

**Fig 9:**
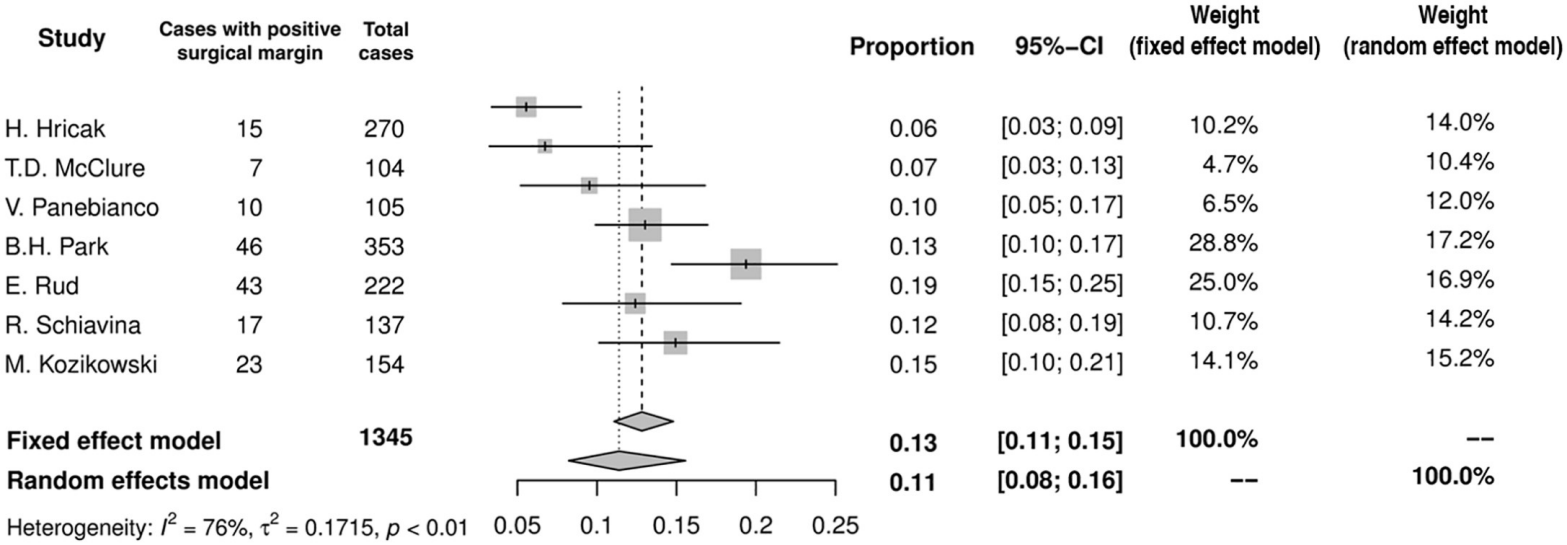
Forest plot of proportions showing overall PSM rate.

Most studies adopted positive surgical margins (PSM) as an adequate oncologic end point indicating the appropriateness of decision based on MRI in individual patients. In total PSM occurred in 11% (95%CI: 8–16%) of men (n = 161; Fig 9). There was no significant difference between groups irrespective of whether the decision was modified (PSM = 14%, 95%CI: 6–29%) based on MRI or remained unchanged (PSM = 15%, 95%CI: 9–23%). Of note, in only 3 studies authors provided results allowing to draw this conclusion [8,12,17].

## Appropriateness of change in surgical plan adaptation

**Fig 10:**
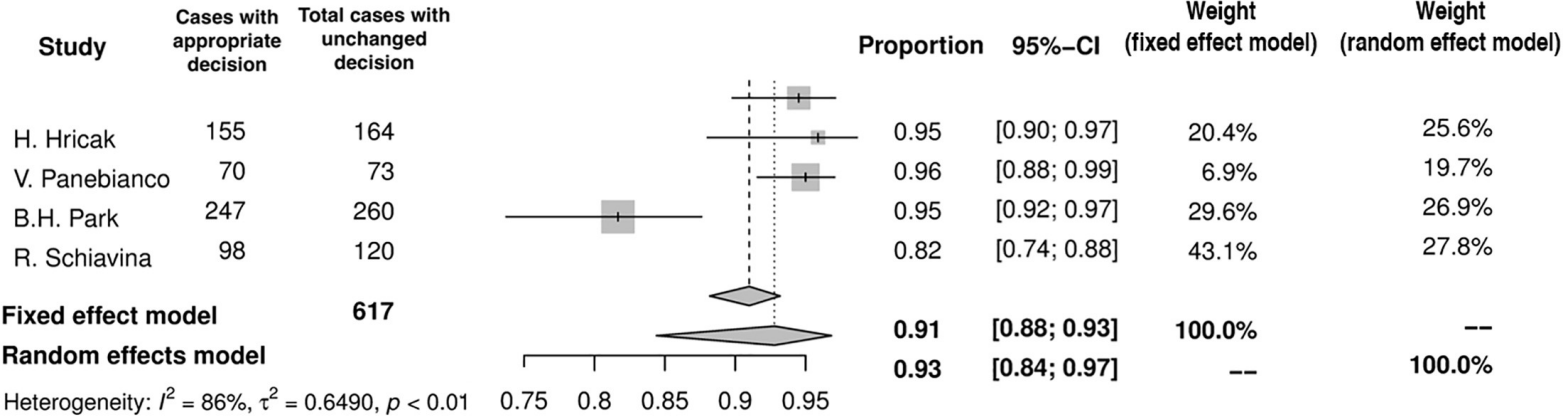
Forest plot of proportions showing appropriateness of unchanged decision.

**Fig 11:**
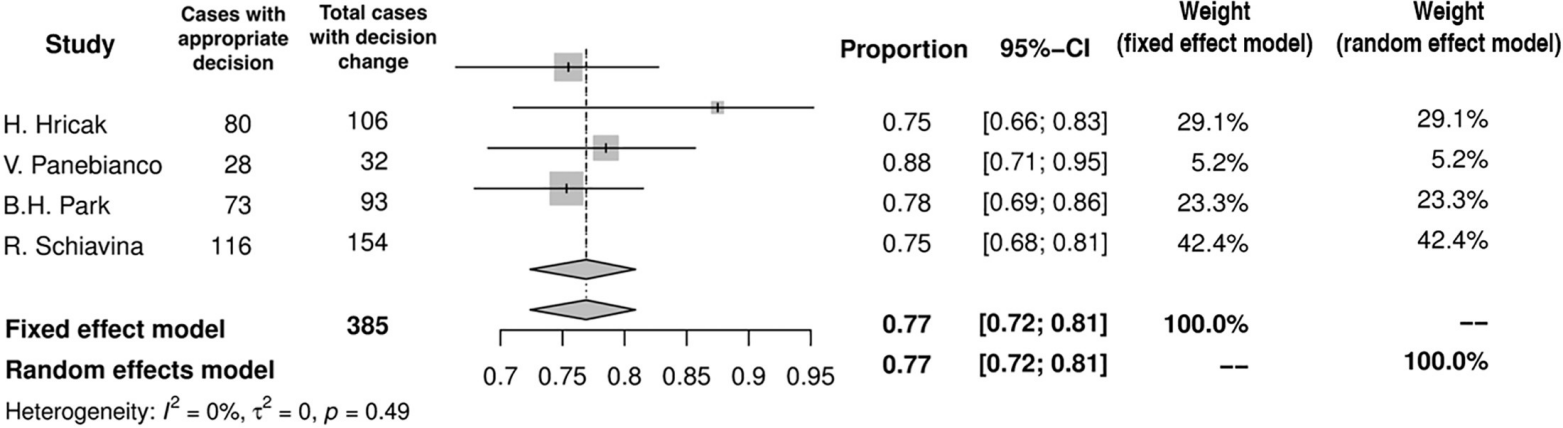
Forest plot of proportions showing appropriateness of changed decision.

In the worst case scenario MRI may misguide a surgeon to unnecessarily remove NVB when dealing with truly organ confined lesion, or conversely, unclear imaging may prompt NVB-sparing in case of truly advanced tumor and lead to PSM. This appropriateness of surgical plan adaptation based on MRI was assessed in 4 studies [10,13,14,16], of which two included subgroup analysis [10,14]. When MRI added no additional information and a surgical template remained unchanged, the decision was correct in 93% (95%CI: 84–97%) of cases (Fig 10). If the surgical template set before MRI was eventually modified after imaging, the appropriateness was 77% (95%CI: 72–81%) (Fig 11). The appropriateness varied among PCa risk groups and amounted to 63% (95%CI: 46–77%) in low-, 75% (95%CI: 60–86%) in inter-, and 91% (95%CI: 80–96%) in high-risk PCa group.

## Discussion

Our systematic review provides updated summary of studies focused on the influence of MRI on the decision-making process prior to RP. The results indicate that the preoperative MRI leads to the modification of initial surgical template in one third of cases. The phenomenon occurs increasingly with the rising PCa risk category: from 28% in low-risk group, through 33% in intermediate-risk group, to 52% in high-risk PCa group. Modification of NVB-sparing surgery based on MRI appeared to have no impact on the PSM rate. The decision based on MRI is appropriate on average in 77% of cases and differs across various PCa risk categories.

### MRI modifies surgical template with respect to NVBs

At present, MRI is considered the most accurate imaging modality in detecting extraprostatic extension (EPE) and provides opportunity for a surgeon to adapt the template for prostate tumor resection [19]. Our meta-analysis has shown preoperative MRI to prompt a change in the decision regarding NVB-sparing in one third (35%) of men underscoring a major role of imaging in decision-making process prior to RP. Intuitively, from the oncological perspective the tendency towards more aggressive resection would be more common than the opposite approach of more preserving surgery. Indeed, across number of studies, the former attitude was observed in 21% of patients, whereas the latter one in 16% of cases. However the rates of adjustments fluctuated across the analysed papers. It was dependent on number of factors including various criteria for nerve-sparing surgery, different MRI specifications (field strengths, MRI sequences, optional endorectal coil, time of MRI) and surgical approaches (open, laparoscopic, robotic) as well (Tables 1 and 2).

Not surprisingly, if preoperative MRI indicates the presence of EPE, one would remove the adjacent bundle regardless of other clinical variables. Lee et al have shown that MRI suggestive of EPE was significantly associated with the excision of NVB [20], and those with concern for extracapsular extension (ECE) on MRI had lower rates of NVB-sparing at least on affected side [20]. At the same time, sensitivities and specificities of MRI in the detection of EPE range from 41% to 92% and from 65% to 100%, respectively (Table 2). These diversities may reflect various accommodation of MRI images in different studies. In the trial by Durskin et al, NVB-sparing rates between MRI and non-MRI group were similar despite suggestion of non-focal EPE in pelvic MRI in selected cases [21]. Therefore, despite fairly high diagnostic accuracy these observations indicate, that MRI before RP is being adopted with great caution. Of note, conservative approach may harm PCa patients being unnecessarily disqualified from the NVB-sparing surgery. Retrospective analysis revealed, that one in four patients (26%) who had their bundles resected because of high-risk disease, could probably have one spared due to MRI that suggested organ-confined disease [15].

Discrepancies in the detection of EPE may also be caused by the lack of standardization in MRI reading. In only three studies included in our systematic review the EPE probability scale was used [12,14,15]. To date, several attempts were made to facilitate standardized PCa staging, with PI-RADS and Likert scale as the most commonly used systems. Despite promising preliminary results [22], the systematic assessment of EPE by points did not gain broader acceptance and was later abandoned in the reissued PI-RADS version 2 recommendations, in which the likelihood of EPE was assessed in a descriptive manner giving a binary result [23]. Even though a structured MRI interpretation is done, results may vary considerably between readers [24,25] and heavily depend on readers experience [26]. These observations highlight the importance of standardization when defining explicit EPE criteria and optimal preoperative planning [27].

Our meta-analysis implies, that incorporation of MRI images into surgical templates is appropriate in 77% of cases. In other words, in around a quarter of patients the decision based on MRI was wrong and resulted in PSM or unnecessary bundle removal. The higher appropriateness of the surgical template, when it remained unchanged (93%), may be explained by that, these were the cases, where MRI findings did not modify initial surgical decision, because it was straightforward due to other clinical variables. Imaging played virtually only a confirmatory role in these cases. For example, it could be the case of low risk PCa without any suspicious lesion visible in MRI, where both NVBs might be preserved. On the contrary, bilateral broad resection would be favored in locally advanced high risk PCa irrespective of the imaging. On the other hand, in more difficult cases, where MRI modified the initial surgical approach, the decision was still made with fairly high accuracy (77%). Unfortunately, the majority of analyzed papers do not provide specific percentages of cases, when MRI led to incorrect decision. However, in the only two studies addressing this issue, MRI-based NVB-sparing procedure was appropriate in most cases (91% and 92%), whereas more aggressive surgery due to MRI turned out to be correct in two-thirds of patients (63% and 60%) [14,16]. These observations indicate misguided tendency towards underestimating the MRI result, when it is negative for EPE, which leads to fewer NVB-preservations.

Lastly, the role of MRI as a prognostic marker has been acknowledged. It has been shown that radiologic findings may serve as a surrogate of adverse pathology after RP or even predict biochemical recurrence and therefore guide towards the optimal choice of cancer treatment [28]. MRI, by visualizing intraprostatic lesions, identify men, who are likely to have clinically significant disease and eventually benefit from radical management [29]. At the same time we have learned from the number of studies including recently published ProtecT trial that PCa specific mortality is very low in low risk PCa irrespective of the mode of therapy. However, among those who prefer active monitoring greater risk of disease progression and metastases has to be taken under consideration [2]. Therefore, men with low risk PCa otherwise eligible for active surveillance may benefit from imaging, which in case of a suspicious result would prompt targeted biopsy and radical treatment. Indeed, abnormal MRI is associated with the greater risk of PCa progression in active surveillance cohorts [30]. The probability of undergrading and understaging is significantly greater in those in whom MRI reveals abnormal lesions when compared to those in whom imaging is normal [29]. In the studies included into our research mean preoperative PSA and Gleason score in biopsy were low, which might suggests that studied cohorts largely embraced men with low-risk disease, yet the rates of locally advanced PCa was substantial (range: 9–43%). Therefore, our results confirm the observation that preoperative assessment of clinical stage without imaging is prone to underestimation [31].

### MRI has no negative impact on the rate of PSM

Incorporation of MRI images into prostatectomy surgical template followed by more restrictive surgery may raise concerns with respect to the status of surgical margins. Ideally adjustment of surgical confines would lead to decrease in the likelihood of PSM with simultaneous increase in NVB preservation. Our meta-analysis has shown that in those who had preoperative MRI rate of PSMs is no different irrespectively of the direction of surgical technique adaptation (Fig 9). In matched control study, despite the difference in crude numbers, PSM rates in pelvic MRI and non-MRI groups were similar (13.7% vs 19.3%) [21]. This relation was also observed at subgroup analysis in patients with T1c PCa (11.0% vs 18.1%) and more advanced disease as well (21.4% vs 25.0%) [21]. In other retrospective study MRI before surgery was not associated with improvement in the rate of PSM [32]. In the only one randomized trial devoted to the role of preoperative MRI, despite advanced imaging overall risk of PSM was not improved (23% in non-MRI vs 19% in MRI group). Subgroup analysis revealed however possible benefit of imaging in patients with T1c cancers only, in whom the relative reduction of PSM by 41% was observed [8]. At the same time, Brown et al. have shown clear trend towards greater risk of PSM in MRI organ confined disease that was found to be locally advanced after prostatectomy. The rate of PSM in this group mounted to 54% [33]. Furthermore, in the lowest risk group category (T1c, GS 6) the corresponding rate reached 80% if understaging based on MRI was encountered [33]. From the other site, multivariable analysis demonstrated that ECE suspected on MRI had neither protective effect nor increased risk for PSM [34]. It seems that erroneous MRI revealing organ confined disease poses a risk of PSM, therefore the accuracy of imaging in local staging should not be overestimated. However, if ECE is suspected, the bundles are to be removed anyway. To conclude, implementation of images as addition to other clinical data into preoperative planning needs to be viewed with caution and the risk of underestimation of real PCa stage with subsequent risk of PSM even in the realm of so advanced technology has to be taken under consideration. Of note, the location of PSMs usually corresponded to the site of the index lesion visualized by MRI, but in one-third of cases PSM was observed in the remote region of the specimen [21]. This finding indicates that, beyond reader’s experience, surgeons’ expertise is also of utmost importance to obtain satisfactory oncologic outcome. The existence of learning curve has been proven for different types of radical prostatectomy and it is reasonable to assume that the minimal number of cases is needed to become proficient. A large multicenter study indicated that after reaching the caseload over 200–250 LRPs the reduction of the PSM rate achieves a plateau [35]. Considering NVB-sparing surgery, the rate of PSM in operated organ-confined tumors is proposed as a credible way to assess outcomes [36]. The precise number of prostatectomies performed by each surgeon were provided in three studies included in our review [8,15,16], precluding meaningful conclusion regarding this issue.

### PCa risk groups and preoperative MRI

Current guidelines do not recommend mpMRI in low-risk patients for local staging, because of insufficient sensitivity in focal EPE detection, unless it is used as a decision tool to select patients for nerve-sparing procedure [1]. The recommendation underscores the need for selecting the most beneficial group of patients, in whom the MRI would influence the decision making-process with respect to the extent of surgery and most desirably reduce the PSM rate. Our meta-analysis indicates, that indeed the relevance of MRI increases with the PCa risk category, yet it is substantial even in the low-risk group.

Some early studies suggested MRI had no incremental value over standard staging approach [10], and low-risk patients would not benefit from preoperative imaging [37]. However, when combined with low prevalence of EPE in the low-risk subgroup, negative predictive value of MRI in staging is high and facilitate right selection of candidates for nerve sparing surgery [9]. It may be explained by low sensitivity of MRI in the detection of EPE [14,17,,37]. EPE is rather unusual phenomenon in low-risk disease, and if it is encountered, it has focal, microscopic character being beyond the scope of MRI resolution [14]. In RCT closely investigating the influence of MRI on the PSM rate, the modification of initial surgical plan was expressed the most in cT3 disease (83%), however it was the cT1 PCa where more radical excision was chosen in 51% of cases and it was the only subgroup, among which MRI reduced significantly the PSM rate [8]. When referring to the current standards in choosing cases for NVB-sparing, the mpMRI is able to modify the extent of surgery in almost every second man (45%) or even more in the low-risk subgroup (63%) [17]. These numbers imply that MRI, in spite of its low sensitivity in detecting EPE in the low-risk PCa group, provides reassurance, that the decision regarding NVBs was correct [14]. However, the level of its appropriateness in this group is at least moderate (63%). It probably results from overstaging and subsequent unnecessary NVB-resection. One may only speculate and weigh the burden of microscopic, focal PSM in MRI understaged PCa against superfluous bundle resection in overstaged disease.

### Limitations

Our study has some limitations. First, most studies included in meta-analysis had retrospective design and as such were subjected to selection bias and were prone to data loss. This is reflected by the moderate risk of bias of the majority of included papers and the heterogeneity of the results in some outcomes of interest. Nevertheless, we believe it resembles current practice of MRI use in decision-making process, which is not yet standardized. Second, only two studies included a control group, which makes impossible to credibly answer, whether MRI brings a benefit in terms of lowering the PSM rate. This issue needs further well-designed studies to be resolved. Third, the number of included studies is limited and some of the subanalyses are based on the low number of patients, therefore the results should be treated with caution. Fourth, studies varied considerably in terms of inclusion criteria for NVB-sparing surgery and interpretation of MRI images. Standardized MRI reading, which becomes the standard of practice was lacking in the majority of studies. As a result, these findings may not be straightforwardly reproduced in other centers.

## Conclusions

In summary, our meta-analysis showed that MRI exerts significant influence on preoperative planning of the extent of resection during RP. This effect may be spotted among different PCa-risk groups. Modification of the NVB-preservation based on the MRI result seems not to influence the PSM rate. Initial findings regarding influence of imaging on oncological outcomes need to be assessed in further studies.

## Supporting information

S1 Table PRISMA checklist. (DOCX)

S2 Table Search strategy. (DOCX)

## References

[1] Sanda MG, Cadeddu JA, Kirkby E, Chen RC, Crispino T, Fontanarosa J, et al Clinically Localized Prostate Cancer: AUA/ASTRO/SUO Guideline, PART I. J Urol. 2017; 10.1016/j.juro.2017.11.095 29203269

[2] Hamdy FC, Donovan JL, Lane JA, Mason M, Metcalfe C, Holding P, et al 10-Year Outcomes after Monitoring, Surgery, or Radiotherapy for Localized Prostate Cancer. N Engl J Med. 2016;375: 1415–1424. 10.1056/NEJMoa1606220 27626136

[3] Bill-Axelson A, Holmberg L, Filén F, Ruutu M, Garmo H, Busch C, et al Radical prostatectomy versus watchful waiting in localized prostate cancer: The Scandinavian prostate cancer group-4 randomized trial. J Natl Cancer Inst. 2008;100: 1144–1154. 10.1093/jnci/djn255 18695132 PMC2518167

[4] Vesely S, Jarolim L, Duskova K, Schmidt M, Dusek P, Babjuk M. The use of early postoperative prostate-specific antigen to stratify risk in patients with positive surgical margins after radical prostatectomy. BMC Urol. 2014;14 10.1186/1471-2490-14-79 25277310 PMC4195911

[5] Salonia A, Burnett AL, Graefen M, Hatzimouratidis K, Montorsi F, Mulhall JP, et al Prevention and management of postprostatectomy sexual dysfunctions part 1: Choosing the right patient at the right time for the right surgery. European Urology. 2012 pp. 261–272. 10.1016/j.eururo.2012.04.046 22575909

[6] Michl U, Tennstedt P, Feldmeier L, Mandel P, Oh SJ, Ahyai S, et al Nerve-sparing surgery technique, not the preservation of the neurovascular bundles, leads to improved long-term continence rates after radical prostatectomy. Eur Urol. 2016;69: 584–589. 10.1016/j.eururo.2015.07.037 26277303

[7] de Rooij M, Hamoen EHJ, Witjes JA, Barentsz JO, Rovers MM. Accuracy of Magnetic Resonance Imaging for Local Staging of Prostate Cancer: A Diagnostic Meta-analysis. Eur Urol. 2016;70: 233–245. 10.1016/j.eururo.2015.07.029 26215604

[8] Rud E, Baco E, Klotz D, Rennesund K, Svindland A, Berge V, et al Does Preoperative Magnetic Resonance Imaging Reduce the Rate of Positive Surgical Margins at Radical Prostatectomy in a Randomised Clinical Trial? Eur Urol. 2015;68: 487–496. 10.1016/j.eururo.2015.02.039 25813692

[9] Samford DM, Hamoen EH, Fütterer JJ, Van Basten JP, Hulsbergen-Van De Kaa CA, Vreuls W, et al The predictive value of endorectal 3 tesla multiparametric magnetic resonance imaging for extraprostatic extension in patients with low, intermediate and high risk prostate cancer. Journal of Urology, 2013;190(5), 1728–1734. 10.1016/j.juro.2013.05.021 23680307

[10] Hricak H, Wang L, Wei DC, Coakley FV, Akin O, Reuter VE, et al The role of preoperative endorectal magnetic resonance imaging in the decision regarding whether to preserve or resect neurovascular bundles during radical retropubic prostatectomy. Cancer. 2004;100: 2655–2663. 10.1002/cncr.20319 15197809

[11] Labanaris AP, Zugor V, Takriti S, Smiszek R, Engelhard K, Nützel R, et al The role of conventional and functional endorectal magnetic resonance imaging in the decision of whether to preserve or resect the neurovascular bundles during radical retropubic prostatectomy. Scand J Urol Nephrol. 2009;43: 25–31. 10.1080/00365590802326610 18759166

[12] McClure TD, Margolis DJA, Reiter RE, Sayre JW, Thomas MA, Nagarajan R, et al Use of MR Imaging to Determine Preservation of the Neurovascular Bundles at Robotic-assisted Laparoscopic Prostatectomy. Radiology. 2012;262: 874–883. 10.1148/radiol.11103504 22274837

[13] Panebianco V, Salciccia S, Cattarino S, Minisola F, Gentilucci A, Alfarone A, et al Use of Multiparametric MR with Neurovascular Bundle Evaluation to Optimize the Oncological and Functional Management of Patients Considered for Nerve-Sparing Radical Prostatectomy. J Sex Med. 2012;9: 2157–2166. 10.1111/j.1743-6109.2012.02794.x 22642466

[14] Park BH, Jeon HG, Jeong BC, Seo S Il, Lee HM, Choi HY, et al Influence of magnetic resonance imaging in the decision to preserve or resect neurovascular bundles at robotic assisted laparoscopic radical prostatectomy. J Urol. 2014;192: 82–88. 10.1016/j.juro.2014.01.005 24440235

[15] Radtke JP, Hadaschik B, Wolf M, Freitag MT, Alt CD, Schwab C, et al The impact of Magnetic Resonance Imaging on prediction of extraprostatic extension and prostatectomy outcome in low-, intermediate- and high-risk Prostate Cancer Patients. Try to find a standard. J Endourol. 2015;29: 1–10. 10.1089/end.2013.0665 26154571

[16] Schiavina R, Bianchi L, Borghesi M, Dababneh H, chessa francesco, Pultrone CV, et al MRI displays the prostatic cancer anatomy and improves the bundles management before robot assisted radical prostatectomy. J Endourol. 2017; end.2017.0701. 10.1089/end.2017.0701 29256639

[17] Kozikowski M, Powroźnik J, Malewski W, Kawecki S, Piotrowicz S, Michalak W et al 3.0-T multiparametric MRI modifies the template of endoscopic, conventional radical prostatectomy in all cancer risk categories. Archives of Medical Science. 2018;14(6):1381–1386. 10.5114/aoms.2018.79006 30393494 PMC6209726

[18] Sterne JAC, Hernán MA, Reeves BC, Savović J, Berkman ND, Viswanathan M, et al ROBINS-I: a tool for assessing risk of bias in non-randomized studies of interventions. BMJ. 2016;355: i4919 10.1136/bmj.i4919 27733354 PMC5062054

[19] Tan N, Margolis DJA, McClure TD, Thomas A, Finley DS, Reiter RE, et al Radical prostatectomy: Value of prostate MRI in surgical planning. Abdominal Imaging. 2012 pp. 664–674. 10.1007/s00261-011-9805-y 21993567

[20] Lee H, Kim CK, Park BK, Sung HH, Han DH, Jeon HG, et al Accuracy of preoperative multiparametric magnetic resonance imaging for prediction of unfavorable pathology in patients with localized prostate cancer undergoing radical prostatectomy. World J Urol. 2017;35: 929–934. 10.1007/s00345-016-1948-6 27738805

[21] Druskin SC, Liu JJ, Young A, Feng Z, Dianat SS, Ludwig WW, et al Prostate mri prior to radical prostatectomy: Effects on nerve sparing and pathological margin status. Res Reports Urol. 2017;9: 55–63. 10.2147/RRU.S128499 28459044 PMC5403124

[22] Boesen L, Chabanova E, Logager V, Balslev I, Mikines K, & Thomsen HS. Prostate cancer staging with extracapsular extension risk scoring using multiparametric MRI: a correlation with histopathology. European Radiology. 2015;25(6), 1776–1785. 10.1007/s00330-014-3543-9 25504428

[23] Weinreb JC, Barentsz JO, Choyke PL, Cornud F, Haider MA, Macura KJ, et al PI-RADS Prostate Imaging—Reporting and Data System: 2015, Version 2. European Urology, 2016;69(1), 16–40. 10.1016/j.eururo.2015.08.052 26427566 PMC6467207

[24] Sonn GA, Fan RE, Ghanouni P, Wang NN, Brooks JD, Loening AM, et al Prostate Magnetic Resonance Imaging Interpretation Varies Substantially Across Radiologists. European Urology Focus. 2018 10.1016/j.euf.2017.11.010 29226826

[25] Shin T, Smyth TB, Ukimura O, Ahmadi N, de Castro Abreu AL, Ohe C, et al Diagnostic accuracy of a five-point Likert scoring system for magnetic resonance imaging (MRI) evaluated according to results of MRI/ultrasonography image-fusion targeted biopsy of the prostate. BJU International, 2018;121(1), 77–83. 10.1111/bju.13972 28749070 PMC6192038

[26] Tay KJ, Gupta RT, Brown AF, Silverman RK, & Polascik TJ. Defining the Incremental Utility of Prostate Multiparametric Magnetic Resonance Imaging at Standard and Specialized Read in Predicting Extracapsular Extension of Prostate Cancer. European Urology, 2016;70(2), 211–213. 10.1016/j.eururo.2015.10.041 26553331

[27] Riney JC, Sarwani NE. Siddique S, & Raman JD. Prostate magnetic resonance imaging: The truth lies in the eye of the beholder. Urologic Oncology: Seminars and Original Investigations, 2018;36(4), 159.e1–159.e5. 10.1016/j.urolonc.2017.12.013 29336979

[28] De Cobelli O, Terracciano D, Tagliabue E, Raimondi S, Bottero D, Cioffi A, et al Predicting pathological features at radical prostatectomy in patients with prostate cancer eligible for active surveillance by multiparametric magnetic resonance imaging. PLoS ONE, 2015;10(10). 10.1371/journal.pone.0139696 PMC4596627 26444548

[29] Schoots IG, Petrides N, Giganti F, Bokhorst LP, Rannikko A, Klotz L, et al Magnetic resonance imaging in active surveillance of prostate cancer: A systematic review. European Urology, 2015;67(4), 627–636. 10.1016/j.eururo.2014.10.050 25511988

[30] Thurtle D, Barrett T, Thankappan-Nair V, Koo B, Warren A, Kastner C, et al Progression and treatment rates using an active surveillance protocol incorporating image-guided baseline biopsies and multiparametric magnetic resonance imaging monitoring for men with favourable-risk prostate cancer. BJU International, 2018;122(1), 59–65. 10.1111/bju.14166 29438586

[31] Pullini S, Signor MA, Pancot M, Zuiani C, Bazzocchi M, Fongione S, & Girometti R. Impact of multiparametric magnetic resonance imaging on risk group assessment of patients with prostate cancer addressed to external beam radiation therapy. European Journal of Radiology, 2016;85(4), 764–770. 10.1016/j.ejrad.2016.01.008 26971421

[32] Padovani G, Anjos G, Guglielmetti G, Franca R, Viana P, Cordeiro M, et al Can magnetic resonance imaging reduce positive surgical margins in radical prostatectomy? Journal of Urology, 2016;195(4), e42 Retrieved from http://www.embase.com/search/results?subaction=viewrecord&from=export&id=L72238861

[33] Brown JA, Rodin DM, Harisinghani M, Dahl DM. Impact of preoperative endorectal MRI stage classification on neurovascular bundle sparing aggressiveness and the radical prostatectomy positive margin rate. Urol Oncol Semin Orig Investig. 2009;27: 174–179. 10.1016/j.urolonc.2008.04.009 18640062

[34] Secin FP, Serio A, Bianco FJ Jr., Karanikolas NT, Kuroiwa K, Vickers A, et al Preoperative and Intraoperative Risk Factors for Side-Specific Positive Surgical Margins in Laparoscopic Radical Prostatectomy for Prostate Cancer. Eur Urol. B. Guillonneau, Department of Urology, Sidney Kimmel Center for Prostate and Urologic Cancers, Memorial Sloan-Kettering Cancer Center, New York, NY, United States; 2007;51: 764–771. 10.1016/j.eururo.2006.10.058 17098356

[35] Secin FP, Savage C, Abbou C, de La Taille A, Salomon L, Rassweiler J, et al The learning curve for laparoscopic radical prostatectomy: An international multicenter study. International Braz J Urol. 2010 10.1590/S1677-55382010000600018 PMC3397250 20952022

[36] Damani A, Van Hemelrijck M, Wulaningsih W, Crawley D, & Cahill D. Are you now a good surgeon? T2 positive margin status as a quality outcome measure following radical prostatectomy. World Journal of Urology, 2017;35(1), 35–43. 10.1007/s00345-016-1836-0 27112152 PMC5233732

[37] Roethke MC, Lichy MP, Kniess M, Werner MK, Claussen CD, Stenzl A, et al Accuracy of preoperative endorectal MRI in predicting extracapsular extension and influence on neurovascular bundle sparing in radical prostatectomy. World J Urol. 2013;31: 1111–1116. 10.1007/s00345-012-0826-0 22249342

