# Supplementary material for: Clinical utility of MRI in the decision-making process before radical prostatectomy: Systematic review and meta-analysis
Source: PLoS One. 2019 Jan 7;14(1):e0210194. doi: 10.1371/journal.pone.0210194 (PMC6322775; doi:10.1371/journal.pone.0210194)
Supplement: S2 Table — (DOCX) [file pone.0210194.s002.docx]

|  | MEDLINE via PubMed |
| --- | --- |
|  | "prostatic neoplasms"[MeSH Terms] OR "prostate cancer”[tiab] OR "pca"[tiab] OR "prostate"[tiab] OR "prostatic"[tiab] |
| AND | "magnetic resonance imaging" [MeSH Terms] OR "multiparametric magnetic resonance imaging"[tiab] OR "multiparametric MRI"[tiab] OR "MRI"[tiab] OR "mpMRI"[tiab] |
| AND | "prostatectomy" [MeSH Terms] OR "radical prostatectomy"[tiab] OR "laparoscopic radical prostatectomy"[tiab] OR "endoscopic radical prostatectomy"[tiab] OR "open radical prostatectomy"[tiab] OR "robot-assissted laparoscopic prostatectomy"[tiab] OR "RP"[tiab] OR "LRP"[tiab] OR "ERP"[tiab] OR "EERP"[tiab] OR "TERP"[tiab] OR "ORP"[tiab] OR "RALP"[tiab] |
|  |  |
|  | Embase via Ovid |
|  | 'prostate cancer':de OR 'prostatic neoplasms':ta,ab OR 'pca':ta,ab OR 'prostate':ta,ab OR 'prostatic':ta,ab |
| AND | 'nuclear magnetic resonance imaging':de OR 'multiparametric magnetic resonance imaging':ta,ab OR 'multiparametric MRI':ta,ab OR 'MRI':ta,ab OR 'mpMRI':ta,ab |
| AND | 'prostatectomy':de OR 'radical prostatectomy':ta,ab OR 'laparoscopic radical prostatectomy':ta,ab OR 'endoscopic radical prostatectomy':ta,ab OR 'open radical prostatectomy':ta,ab OR 'robot-assissted laparoscopic prostatectomy':ta,ab OR 'RP':ta,ab OR 'LRP':ta,ab OR 'ERP':ta,ab OR 'EERP':ta,ab OR 'TERP':ta,ab OR 'ORP':ta,ab OR 'RALP':ta,ab |
|  |  |
|  | Cochrane Database of Systematic Reviews |
| #1 | MeSH descriptor: [Prostatic Neoplasm] explode all trees |
| #2 | 'prostate cancer':de or 'pca':ti,ab or 'prostate':ti,ab or 'prostatic':ti,ab |
| #3 | #1 or #2 |
| #4 | MeSH descriptor: [Magnetic Resonance Imaging] explode all trees |
| #5 | 'multiparametric magnetic resonance imaging':ti,ab or 'multiparametric MRI':ti,ab or 'MRI':ti,ab or 'mpMRI':ti,ab |
| #6 | #4 or #5 |
| #7 | MeSH descriptor: [Prostatectomy] explode all trees |
| #8 | 'radical prostatectomy':ti,ab OR 'laparoscopic radical prostatectomy':ti,ab OR 'endoscopic radical prostatectomy':ti,ab OR 'open radical prostatectomy':ti,ab OR 'robot-assissted laparoscopic prostatectomy':ti,ab OR 'RP':ti,ab OR 'LRP':ti,ab OR 'ERP':ti,ab OR 'EERP':ti,ab OR 'TERP':ti,ab OR 'ORP':ti,ab OR 'RALP':ti,ab |
| #9 | #7 or #8 |
| #10 | #3 and #6 and #9 |
|  |  |
| Note: | The search in all databases was restricted to publications in English, dating from January 2000 to April 2018 |
